# Specialized Pro-resolving Lipid Mediators and Glial Cells: Emerging Candidates for Brain Homeostasis and Repair

**DOI:** 10.3389/fncel.2021.673549

**Published:** 2021-04-26

**Authors:** Marta Tiberi, Valerio Chiurchiù

**Affiliations:** ^1^Laboratory of Resolution of Neuroinflammation, European Center for Brain Research, IRCCS Santa Lucia Foundation, Rome, Italy; ^2^Institute of Translational Pharmacology, National Research Council, Rome, Italy

**Keywords:** inflammation, resolution, specialized pro resolving mediators, lipids, microglia, macroglia

## Abstract

Astrocytes and oligodendrocytes are known to play critical roles in the central nervous system development, homeostasis and response to injury. In addition to their well-defined functions in synaptic signaling, blood-brain barrier control and myelination, it is now becoming clear that both glial cells also actively produce a wide range of immune-regulatory factors and engage in an intricate communication with neurons, microglia or with infiltrated immune cells, thus taking a center stage in both inflammation and resolution processes occurring within the brain. Resolution of inflammation is operated by the superfamily of specialized pro-resolving lipid mediators (SPMs), that include lipoxins, resolvins, protectins and maresins, and that altogether activate a series of cellular and molecular events that lead to spontaneous regression of inflammatory processes and restoration of tissue homeostasis. Here, we review the manifold effects of SPMs on modulation of astrocytes and oligodendrocytes, along with the mechanisms through which they either inhibit inflammatory pathways or induce the activation of protective ones. Furthermore, the possible role of SPMs in modulating the cross-talk between microglia, astrocytes and oligodendrocytes is also summarized. This SPM-mediated mechanism uncovers novel pathways of immune regulation in the brain that could be further exploited to control neuroinflammation and neurodegeneration.

## Resolution of Neuroinflammation

Inflammation is a self-limited and protective process that usually resolves on its own and restores tissue homeostasis. Occurrence of inflammation within the central nervous system (CNS) is referred to as neuroinflammation, and similar to what happens for the rest of body districts and tissues, when persistent or unresolved is detrimental to neurological functions and leads to neurodegeneration (Nathan and Ding, 2010). Contrary to what has been believed for over a century, the CNS is not an immuno-privileged tissue and neuroinflammatory events occur in the brain parenchyma leading to glial cell activation and recruitment of leukocytes from the periphery to survey for CNS self-antigens or to fight potential pathogens or aggregated proteins (Schwartz and Baruch, 2014). Although the mechanisms that cause neurodegenerative diseases are different, neuroinflammation is a common hallmark and only recently the interactions between the CNS and circulating immune cells are becoming more fully understood, also thanks to the recently identified neuro-immunological interfaces that serve as gateways to allow immunosurveillance and that, when altered or disrupted, can lead to pathological conditions (Da Mesquita et al., 2018a,b; Rustenhoven et al., 2021).

This evidence not only confirms the “protective autoimmunity” theory that was proposed more than 20 years ago as an essential physiologic mechanism for CNS protection, repair and maintenance in both health and disease (Moalem et al., 1999), but also implies that the concept of resolution of inflammation, together with the underlying temporal lipid mediator class switch and production of the superfamily of specialized pro-resolving lipid mediators (SPMs), holds true also in the CNS for the restoration of full tissue homeostasis and the avoidance of neurodegeneration.

**FIGURE 1:**
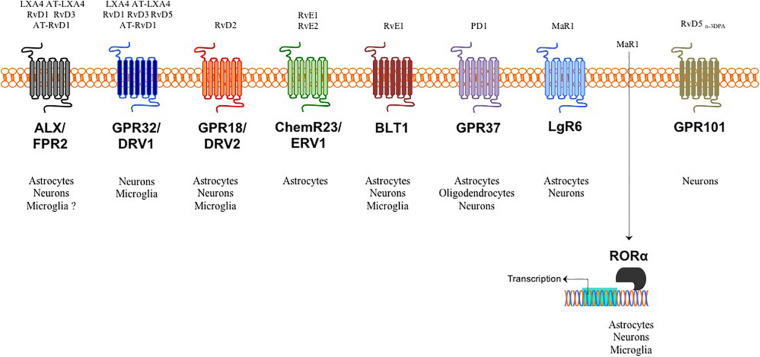
SPMs, target receptors and their cellular distribution in the brain. Target receptors for LXA4, RvD1, RvD3, RvD5 and some of their aspirin triggered analogs bind to either astrocytic and neuronal ALX/FPR2 in both humans and mice or to neuronal and microglial GPR32/DRV1 in humans. RvD2 target receptor is GPR18/DRV2, which is distributed in all brain cells except oligodendrocytes. RvE1/2 activate the pro-resolving ChemR23/ERV1 receptor on astrocytes and inhibit the pro-inflammatory BLT1 receptor in astrocytes, microglia and neurons. The target receptor for PD1 is GPR37 expressed in all brain cells except microglia. MaR1 can bind and activate both surface LgR6 in astrocytes and neurons and intracellular RORα expressed in all brain cells except oligodendrocytes. As yet, only neurons express the RvD5_n–3D__PA_ receptor.

Indeed, during resolution of inflammation, the very same cells that are involved in the first steps of acute inflammation (i.e., vascular endothelial cells and innate immune cells) gradually stop to produce the highly pro-inflammatory classical eicosanoids (prostaglandins, leukotrienes, thromboxanes) from ω-6 arachidonic acid (AA) and begin to utilize ω-3 eicosapentaenoic acid (EPA) and ω-3 docosahexaenoic acid (DHA) to generate over 30 different SPMs (Serhan, 2014; Basil and Levy, 2016; Chiurchiù et al., 2018). These include different families such as resolvins, protectins and maresins and that, although characterized by different biosynthetical pathway and structure, they all share the common feature of activating the signs of resolution: removal, relief, restoration, regeneration, and remission (Serhan, 2014; Basil and Levy, 2016; Serhan et al., 2020). The recent evidence that different cells of the CNS express several SPM receptors (Figure 1) suggest that the activation of pro-resolution pathways are also occurring in the brain and that SPMs might be critical in modulating the crosstalk between glial cells and neurons and thus in affecting the initiation and progression of several neuropathologies.

### AA-Derived Lipoxins

AA is an ω-6 PUFA presents in all human tissues and it is esterified to membrane phospholipids at the sn-2 position. Upon inflammation, phospholipase A2 cleaves free AA which is then metabolically converted in a myriad of pro-inflammatory mediators, generally termed eicosanoids or oxylipins. These include mainly prostaglandins, leukotrienes and thromboxanes, which are the classical mediators of inflammation and the responsible of the five cardinal signs of inflammation: redness, heat, swelling, pain, and loss of function.

However, during the peak of inflammation AA can further be metabolized into Lipoxins (LXs), which belong to the superfamily of SPMs and comprise only two isomers: LXA_4_ and LXB_4_, which were first described in 1984 by Serhan, Hamberg and the Nobel Laureate Samuelsson (Serhan et al., 1984). The biosynthetic pathway of LXs is a transcellular process that occurs between inflammatory cells (usually eosinophils, macrophages, and dendritic cells) and resident cells in the inflammatory locus (i.e., endothelial cells), and it proceeds through three different biochemical routes, each one characterized by two consecutive oxygenation steps (Bannenberg and Serhan, 2010). The first biochemical route involves the catalysis of AA by 15 lipooxygenase (15-LOX) in endothelial cells, monocytes or eosinophils, generating the 15-S-HpETE intermediate, which is then converted in 5,6-epoxytetraene by 5-LOX before finally being hydrolyzed into either LXA_4_ or LXB_4_ (Bennet and Gilroy, 2016). The second synthesis passes through AA oxygenation into leukotriene A4 (LTA_4_) and proceeds toward LXA_4_ and LXB_4_ conversion via 12-LOX (Freire and Van Dyke, 2013). Lastly, a third biosynthetic pathway involves administration of aspirin, a commonly used non-steroidal anti-inflammatory drug which triggers irreversible acetylation of cyclooxygenase-2 (COX-2), the enzyme responsible for AA conversion into prostaglandins. Blockage of COX-2 activity allows formation of 15-R-HETE intermediate that is rapidly released and captured by adherent leukocytes or vascular endothelial cells and further metabolized by 5-LOX to form 15-epimeric-LXs or aspirin-triggered LXs (AT-LXs) (Serhan, 2005).

LXA_4_, LXB_4_ and AT-LXs are all components of the LXs class of SPMs and they share the same anti-inflammatory and pro-resolving functions, which include to stimulate cellular and tissue responses that trend to reverse the actions of the pro-inflammatory mediators, dampen and reverse the inflammatory response, and initiate tissue repair. These effects include the uptake of apoptotic polymorphonuclear cells by macrophages, the removal of cell debris and pathogen killing (Serhan, 2014; Romano et al., 2015) as well as immunomodulation by targeting innate and adaptive immune cells and the reduction of oxidative stress (Ariel et al., 2006; Leuti et al., 2019; Kooij et al., 2020). The mechanism of action of LXs is exerted by the formyl peptide receptor 2 (FPR2), also known as ALX. This G protein-coupled receptors (GPCR) is bound by LXA_4_ but also shared by others SPMs (Chiang et al., 2000, 2005). LXs were the first components of SPMs recognized as endogenous lipid mediators in mediating resolution of inflammation and are often referred to as the initiators of this biological process and the trigger of the metabolic switch (Serhan, 2001). To date, no specific receptor for LXB_4_ has been identified yet.

### EPA Derived E-Series Resolvins

During the metabolic switch, occurring at the peak of an inflammatory event, the very same cells that generated the classical eicosanoids or lipoxins, start to utilize the ω3 EPA as a substrate instead of AA. EPA is thus the precursor of the SPM class of E-series resolvins (RvEs). The biosynthesis of RvEs is initiated by vascular endothelial cells that convert EPA into 18-HEPE and proceeds via a transcellular biosynthesis via acetylated COX-2 and cytochrome P450. Subsequently leukocytes through 5-LOX and a series of enzymatic epoxidation and hydrolysis generate RvE1 (Serhan et al., 2000; Arita et al., 2005). During this multi-step biochemical route, also RvE2 can be generated (Tjonahen et al., 2006). Of note, 18-HEPE can directly be transformed into RvE3 through the action of 12/15-LOX by several leukocytes (Isobe et al., 2012). This year, a new member of the EPA–derived E-series resolvins, RvE4, was elucidated and its stereochemistry was assigned; its production is operated in physiologic hypoxia by human neutrophils and macrophages by 5-LOX into 15S-HpEPE, which can undergo a second enzymatic lipoxygenation to yield a hydroperoxyl group at the carbon C-5 position, which is reduced to form RvE4 (Libreros et al., 2021).

RvE1-E4 are more prominently involved in reducing inflammation by increasing efferocytosis of apoptotic cells by macrophages, blocking leukocyte recruitment into inflamed tissues, to modulate inflammatory responses of key immune cells and also to reduce pain (Vik and Hansen, 2021). The pro-resolving actions of RvE1 and RvE2 are due to their ability to activate ChemR23 and to antagonize BLT1, which usually mediates the proinflammatory actions of LTB_4_ (Chiang and Serhan, 2017). Both receptors are widely expressed receptors in several cells in the periphery and in the CNS. To date, the receptors for RvE3 and RvE4 remain to be identified.

### DHA Derived E-Series Resolvins, Protectins, and Maresins

EPA through Elongase 5 is metabolically converted into docosapentanoic acid (DPA), which can also be converted into n-3 DPA series resolvins, protectins, and maresins (Dalli et al., 2013; Hansen et al., 2017) and is also the precursors of 13-series or T-resolvins (RvT1-4) (Dalli et al., 2015). As yet, besides the role of RvD5n-3DPA and its receptor GPR101 in mediating resolution of inflammatory arthritis and pathogen infection (Flak et al., 2020), the biological role of DPA-derived SPMs is yet to be fully elucidated. However, DPA is quickly converted into DHA by means of Elongase 2 and a desaturase (Talamonti et al., 2020) and the majority of SPMs originate from this ω-3 fatty acid, which can be converted into D-series Resolvins (RvDs), Protectins (PDs), and Maresins (MaRs) as well as into their more recently identified sulfido-conjugates. Each group is made up of multiple molecules, altogether comprising more than 20 biologically active SPMs.

D-series resolvin synthesis starts off by 15-LOX-mediated conversion of DHA into 17(S)-hydroperoxy DHA (17(S)-HpDHA). From this, the fate of 17(S)-HpDHA depends on 5-LOX action and on which carbon is hydroxylated by its catalytic action. In fact, it can be converted either into 7(S)-hydroperoxy-17(S)-HDHA to generate RvD1, RvD2, and RvD5 or into 4(S)-hydroperoxy- 17(S)HDHA to generate RvD3, RvD4, and RvD6. Alternatively, 17(S)-HpDHA can be converted to a 16,17-epoxydocosatriene intermediate that in turn is converted to protectin D1 (or neuroprotectin D1 when produced in brain tissues) and PDX. Similarly to E-series resolvins, aspirin-acetylated COX-2 can use the D-resolvin precursor, DHA, to generate 17(R)-HpDHA, which is then transformed to epimeric AT-D-resolvins or AT-protectins in a 5-LOX-dependent manner (Serhan, 2014; Serhan et al., 2015). The last family of SPMs belonging to DHA-derived SPMs was discovered in macrophages, therefore named maresins. The biosynthesis of maresins (MaR1 and MaR2) is operated by 12-LOX in humans and by 12/15LOX in mice from 14(S)-HDHA intermediate, which is recognized as marker of maresins (Serhan et al., 2009; Deng et al., 2014).

The bioaction of these 3 classes of DHA-derived SPMs are shared in common with those of the other SPMs because they reduce leukocyte infiltration, induce pathogen killing, promote the clearance of debris and dead cells by the process of efferocytosis and reduce production of pro-inflammatory mediators and enhance that of anti-inflammatory ones. These biological activities are mediated by several receptors with different affinities to different SPMs of this class. For instance, several D-series resolvins (i.e., RvD1, RvD3, and RvD5) bind to two different GPCRs, FPR2/ALX and GPR32/DRV1, whereas RvD2 engages GPR18/DRV2 receptor (Chiang and Serhan, 2017). In the last couple of years, the molecular targets for PD1 and MaR1 have also been uncovered and these two SPMs, respectively, bind to surface receptors GPR37 (Bang et al., 2018) and LgR6 (Chiang et al., 2019), although the latter SPM can also engage with the nuclear receptor RORα (Han et al., 2019).

Interestingly, starting from 2014 it was discovered that all these 3 families of DHA-derived SPMs can be biochemically conjugated to glutathione and give rise to a series of novel mediators coined “conjugates in tissue regeneration” and that include mares in conjugates in tissue regeneration (MCTRs), protectin conjugates in tissue regeneration (PCTRs), and resolvin conjugates in tissue regeneration (RCTRs) (Dalli et al., 2016; Serhan et al., 2018). As suggested by their name, their physiological function is to orchestrate host responses and promote tissue regeneration and resolution of infections and has been demonstrated in different model organisms (Serhan et al., 2018).

## Spms and Glial Cells

Since the discovery of the different families of SPMs, their role was mostly investigated in peripheral tissues mainly because, to date, their production is carried out by innate immune cells (i.e., granulocytes and macrophages) or vascular endothelial cells. However, in the last decade, on the verge of the revisitation of the concept of the CNS immune privilege and the realization that a network of infiltrated immune cells operates within the brain in a tightly regulated sequence of inflammation/resolution processes, several groups started to investigate whether SPMs are also capable of modulating neuroimmune functions by targeting either neurons or glial cells. Furthermore, recent evidence show that altered SPM metabolism and function is associated to several neuroinflammatory and neurodegenerative diseases, such as Alzheimer’s disease, Parkinson’s disease, and multiple sclerosis (Chiurchiù et al., 2018; Chamani et al., 2020; Zahoor and Giri, 2020).

### SPMs and Astrocytes

Among all glial cells, astrocytes are probably the cell type that fits the most with the concept of resolution of inflammation due to their vital role in regulating CNS homeostasis. They are by far the most abundant cells in the CNS, where they regulate virtually every physiological process, including: BBB maintenance, neurotrophin secretion, and regulation of neuronal synaptogenesis and elimination, ion buffering, neurotransmitter recycling, synaptic plasticity and modulation of immune functions (Miller, 2018). Recent evidence suggest that these functions are performed in a diverse fashion by different subsets of astroglia. Similarly to macrophages/microglial cells, astrocytes were recently classified into neurotoxic A1 and neuroprotective A2 cells; however, such nomenclature is to be taken with a grain of salt and it seems that these cells display a high degree of heterogeneity during neuroinflammation. However, only a limited body of literature describes morphological and functional changes of astrocytes along the full spectrum of activation stages and also during the progression of neurodegenerative diseases (Zhang and Barres, 2010). In this context, it’s indisputable that immune activation of astrocytes in response to signals released by injured neurons or activated microglia leads to the so-called astrogliosis. Astrogliosis, a hallmark of neuroinflammation, is characterized by a higher production of pro-inflammatory cytokines and reactive species and a lower production of neurotrophic factors, determining glial scars that prevent axonal regeneration and that represents a feature of many neurodegenerative diseases (Jensen et al., 2013; Ding et al., 2021).

Several works reported that astrocytes express high levels of ALX/FPR2 both *in vivo* and *in vitro* and in several brain areas (Svensson et al., 2007; Cattaneo et al., 2010; Bisicchia et al., 2018). Such expression seems to be particularly evident in the spinal cord, with a homogenous distribution in both dorsal and ventral horns (Svensson et al., 2007). This was also confirmed *in vitro* in several astrocytoma cell lines, that expresses a functional receptor both at mRNA and protein level (Le et al., 2000; Decker et al., 2009). In these cells, LXA_4_, one of the SPMs that efficiently binds this receptor, has an inhibitory effect on the expression of the proinflammatory chemokine IL-8 and adhesion molecule ICAM-1 in response to IL-1β and via an NF-kB-dependent mechanism (Decker et al., 2009). Interestingly, ALX/FPR2 was reported to be involved in the internalization and subsequent removal of Aβ42 in inflamed astrocytes through a physical interaction with the scavenger receptor MARCO (Brandenburg et al., 2010). This receptor has also been linked to MAPK and the inflammatory response in astroglioma U-87 cells, where its activation with its highly potent agonist WKYMVm induces JNK and ERKs phosphorylation and increases the expression of glial fibrillary acidic protein (GFAP) and IL-1β, which are correlated with astrogliosis (Kam et al., 2007). Of note, the involvement of ALX/FPR2 in the regulation of astrogliosis has major biological implications, because reactive astrocytosis and brain inflammation are pathological features of many neuroinflammatory and neurodegenerative diseases.

Indeed, it was reported that LXA_4_ and RvD1, the two major SPMs to bind this receptor, reduce astrocyte reactivity by inhibiting their activation and/or pro-inflammatory cytokines production (Svensson et al., 2007; Ren et al., 2020) and by protecting neurons, demonstrating an astrocyte-dependent neuroprotective activity (Ren et al., 2020). Additionally, in a mouse model of postoperative cognitive dysfunction, acute administration of AT-RvD1 prevented neuronal dysfunction and cognitive impairment by regulating long-term potentiation, and astrocyte activation (Terrando et al., 2013). Our group also recently found that peripheral RvD1 administration in a focal brain injury model improved functional recovery, protected neurons from cell death and reduced activation of microglial cells in a mechanism that was dependent on the activation of an anti-inflammatory ALX/FPR2-regulated microRNA pathway probably induced by astrocytes (Bisicchia et al., 2018).

The finding that ALX/FPR2 is highly expressed in spinal astrocytes is intriguing, since increasing evidence indicates that non-neuronal cells play an important role in spinal facilitation of pain processing. Indeed, not only it has been shown that both nerve injury and peripheral inflammation lead to activation of spinal dorsal horn astrocytes (Sweitzer et al., 1999), but also that spinal delivery of inhibitors or modulators of astrocyte function or administration of lipoxins block initiation and maintenance of persistent pain states (Zhuang et al., 2006; Svensson et al., 2007), supporting an important role for these cells in spinal sensitization and suggesting that the lipoxin/ALX pathway regulates astrocyte-dependent spinal nociception.

Furthermore, astrocytes express also the RvE1 receptors ChemR23/ERV1 in human hippocampus (Wang et al., 2015) and BLT1, the pro-inflammatory receptor that is blocked by RvE1, in both the gray and white matter of several brain regions, including frontal cortex, forebrain, cerebellum and hippocampus (Emre et al., 2020). However, another work reported that BLT1 was mainly expressed in neurons, microglia and endothelial cells but not in astrocytes (Ye et al., 2016).

On account of the fact that ChemR23 might be indeed expressed on astrocytes and it bears a protective role, a recent study performed a chronic and peripheral treatment with RvE1 and LXA_4_ alone or in combination in a mouse model of Alzheimer’s disease and found that the combined treatment potently reduced astrocyte activation in both hippocampus and cortex and ameliorated AD pathology and the production of several cytokines and chemokines (Kantarci et al., 2018). Of note, all these results were obtained in mice where SPMs were administered peripherally and suggest that resolvins are indeed able to cross the blood–brain barrier and exert their effect in the brain.

Interestingly, cortical astrocytes also express high levels of GPR37 (Galey et al., 2003; Cahoy et al., 2008) and its activation with a potent agonist significantly protected them from oxidative stress-induced death, with these protective effects being attenuated by siRNA-mediated knockdown of endogenous astrocytic GPR37 (Meyer et al., 2013). However, the binding activity of previously used agonists on GPR37 was recently challenged, making this receptor to regain an orphan status until 2018, when it was first confirmed the truthfulness of the initial binding data and its neuroprotective effect from oxidative stress (Liu et al., 2018) and then the identification of PD1 as the endogenous ligand (Bang et al., 2018). However, in the same year another study showed that astrocytes express low levels of GPR37, which was mainly found in oligodendrocytes, but high levels of homologous GPR37-like 1, which played a critical role in protecting neurons during ischemia by modulating astrocyte glutamate transporters and neuronal NMDA receptors (Jolly et al., 2018).

Although GPR32 seems to be completely absent in astrocytes, these cells express also GPR18 (Grabiec et al., 2019) and even the more recently identified LgR6 (Zhang et al., 2014; Miller et al., 2019). In both cases, the role of these two receptors in astrocytes was studied by stimulating them not with their specific SPMs but with the endocannabinoid N-arachidonoyl glycine for GPR18 and with R-spondin for LgR6 and both showed an astrocyte-dependent protective effects on neurons (Zhang et al., 2014; Miller et al., 2019), highlighting the key role of astrocytes in controlling neuronal functions.

### SPMs and Oligodendrocytes

Oligodendrocytes are the second most abundant population of the CNS and their association with the concept of resolution of inflammation and tissue repair is due to their main role in producing myelin not only in physiological conditions but also in self-resolving situations where myelin loss occurs and is continuously repaired. However, a persistent neuroinflammation often leads to a progressive failure of these cells to restore myelin sheaths (Stadelmann et al., 2019). This is because they are the end product of a cell lineage (i.e., oligodendrocyte precursor cells, OPCs) which has to undergo a complex and spatially and temporally regulated program of proliferation, migration, differentiation, and myelination to finally produce the insulating sheath. Due to this complex differentiation program and their unique metabolism/physiology, oligodendrocytes are considered among the most vulnerable cells of the CNS and put them at a greater risk of damage during neuroinflammation and during pathological conditions (Bradl and Lassmann, 2010). Interestingly, from an immunological point of view, oligodendrocytes were originally thought of as inert and merely representing bystander victims of immune responses. This view has now changed in the light of accumulating evidence that oligodendrocytes actively produce a wide range of immune-regulatory factors (IL-1β, IL-6, IL-8, CCL2) and express immune markers or receptors for such factors (COX-2, MHC-I, CD200, pattern recognition receptors and cytokines/chemokine receptors) (Peferoen et al., 2014), in turn shaping the immune response of other glial cells (Liu and Aguzzi, 2020; Boccazzi et al., 2021). Of note, the presence of COX-2 in these cells and given the contribution of this enzyme to the production of also E-series resolvins, it’s likely that oligodendrocytes might also exert immunomodulatory and homeostatic functions on neurons or other glial cells through SPM production.

In keeping with the information collected till now, among all SPM receptors identified so far, oligodendrocytes seem to express only GPR37 both in the brain and spinal cord, where it plays a critical role in the differentiation of these glial cells and in myelin production (Yang et al., 2016). Although Schwann cells of the peripheral nervous system stained positively for ALX/FPR2, oligodendrocytes were negative for this receptor (Cattaneo et al., 2010).

PD1 is considered a ligand for GPR37 because it induced a significant increase in intracellular calcium in HEK293 cells overexpressing GPR37 and in murine macrophages (Bang et al., 2018). Although in this last work the authors report that GPR37 is expressed in macrophages but not microglia, due to its positive colocalization with CD68 and lack of colocalization with CX3CR1 or Iba1, these data need to be better confirmed since all these three markers are expressed by both monocytes/macrophages and microglial cells, therefore they cannot be used to distinguish the two cell types.

Interestingly, GPR37, also known as Parkin-associated endothelin-like receptor (Pael-R) was originally discovered through genomic library screening to find new neuropeptide receptors (Marazziti et al., 1997) and is associated with neurological disorders, such as Parkinson’s disease (PD), and autism (Lopes et al., 2015). Mutations within the GPR37 gene affect a variety of autism spectrum disorders (Fujita-Jimbo et al., 2012), dopamine reuptake regulation (Marazziti et al., 2007), oligodendrocyte differentiation (Yang et al., 2016), and demyelination (Smith et al., 2017). Thus, although, so far, no evidence is reported on the potential role of SPM in regulating oligodendroglial functions, this evidence suggests that PD1 might be the ideal candidate to test on these cells and especially on demyelinating diseases and PD.

### SPMs in the Cross-Talk Between Microglia, Other Glial Cells, and Neurons

Differently to astrocytes and oligodendrocytes that establish strict contact with neurons, providing them protection and support (Amaral et al., 2016; Verkhratsky and Nedergaard, 2018), microglia are the immune sentinels of the CNS and are critical for its development and surveillance (Ransohoff and Cardona, 2010; Prinz et al., 2019). To do so, microglial cells are extremely heterogeneous because they can exist in many different forms and activation states, from neuro-protective to neuro-destructive (Nayak et al., 2014). According to this, progression or resolution of neuroinflammation or of CNS disorders is strictly relying on the activity of microglia and, being immune cells, it’s no surprise that most of the literature on SPM function in the CNS has been focused on this cell subtype. Indeed, not only they seem to express all the identified SPM receptors but also are highly responsive to the pro-resolving and anti-inflammatory effects of several families of SPMs, from lipoxins, E-series and D-series resolvins to protectins and maresins.

These SPMs have been found to strongly reduce microglial activation, their production of pro-inflammatory mediators and morphology and to increase their ability to phagocytize and remove aggregated proteins such as Aβ, as vastly reported in several comprehensive reviews (Chiurchiù et al., 2018; Dokalis and Prinz, 2019). The importance of their role was also reported in several models of neurodegenerative diseases, such as Alzheimer’s disease (Wang et al., 2015), multiple sclerosis Poisson et al., 2015), and Parkinson’s disease (Krashia et al., 2019).

Furthermore, being DHA the most abundant fatty acid in the human brain and although microglia express several enzymes responsible for SPM biosynthesis (i.e., COX-2, 5-LOX, 12-LOX, and 15-LOX), thus having what it takes to be the main cellular source of SPMs within the CNS, so far, only few studies have analyzed the cellular origin of SPMs in the brain. For instance, *in vitro* studies demonstrated production of PD1 in mixed human neuron-glia cell cultures (Lukiw et al., 2005) and on the human microglia cell line CHME-3 showed LXA_4_ and RvD1 in the conditioned medium (Zhu et al., 2015). Furthermore, immunohistochemical studies on human and murine brains have shown the occurrence of the biosynthetic enzymes 5-LOX and 15-LOX in neurons and glia (Ohtsuki et al., 1995; Lammers et al., 1996; Pratico et al., 2004; Ikonomovic et al., 2008; Haynes and van Leyen, 2013). However, despite lack of evidence on SPM production from neurons or oligodendrocytes, a recent study performed a metabolomic screening on retinal astrocytes and, using liquid chromatography–tandem mass spectrometry, detected pathways markers for DHA-derived resolvins and protectins as well as the two SPMs LXA_4_ and LXB_4_ and that their levels are reduced following injury. This suggests that the lipoxin circuit, comprising biosynthetic enzymes and the ALX/FPR2 receptor, is present in astrocytes and that its functional activity is compromised in response to injury (Livne-Bar et al., 2017). In this study it was also reported that such astrocyte-derived lipoxins promoted neuroprotection in a chronic model of glaucoma, identifying a potential paracrine mechanism that coordinates neuronal homeostasis and inflammation in the CNS.

**FIGURE 2:**
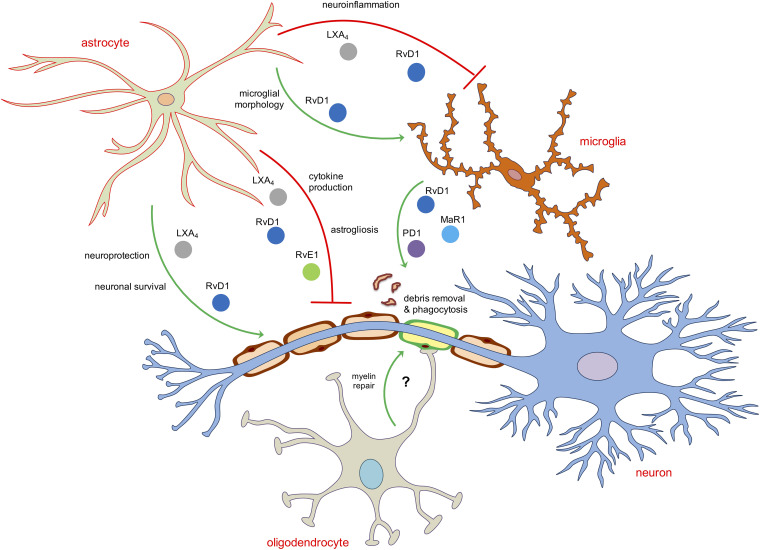
Schematic representation of the main role of SPMs in modulating neuroinflammatory and pro-resolving functions of astroglia and oligodendroglia. Astrocytes and microglia are the two main direct targets of SPMs. LXA_4_, RvD1 and RvE1 inhibit astrocytic and microglial reactivity mainly by reducing pro-inflammatory cytokines or shaping their morphology as well as induce neuroprotection and neuronal survival acting indirectly on astrocytes. The removal of debris, dead cells or aggregated proteins in the brain is operated mainly by RvD1, MaR1 and PD1 by enhancing the phagocytic activity of microglial cells. No direct role of SPMs on oligodendrocytes has been reported yet.

These evidences account for the existence of a crosstalk between glial cells and neurons that is not only limited to the synapses or to the release of inflammatory mediators or neurotrophic factors but also on the activation of pro-resolution pathways that involve the production of SPMs, most likely from microglia and astrocytes, that exert autocrine or paracrine activity on the resolution of inflammatory events or injury occurring to the neurons or to oligodendrocytes, in order to maintain CNS homeostasis and to sustain reparative processes (Figure 2).

## SPMs as Novel Tools to Modulate Neuro-Glial Communications

In addition to classical means of cell-to-cell communication, the transfer of extracellular vesicles (EVs) from microglia, astrocytes or oligodendrocytes to surrounding cells has been recently proposed to be an efficient mechanism by which these cells may contribute to sustain brain homeostasis, immune functions and tissue regeneration. Indeed, not only glia and neurons can secrete EVs exerting a profound impact in CNS pathophysiology, but brain-derived EVs can also travel further through the body via the CSF and blood and thus impact also peripheral districts (Prada et al., 2013; Lizarraga-Valderrama and Sheridan, 2021). Independently of their cell origin and in comparison with their originating cells, EVs are enriched in membrane phospholipids containing saturated and unsaturated fatty acids.

Since SPMs are indeed originated from such membrane phospholipids, EVs may also represent sources of these lipid mediators either already formed or partially synthesized by their biosynthesizing enzymes packaged within vesicles. Conveying SPMs via EVs represents a mean to protect these mediators from degradation and exchange these molecules between different cell types (Sagini et al., 2018). Indeed, through a systematic lipidomic profiling of EVs isolated from inflammatory exudates during the time course of a self-resolving inflammation, several SPM precursors such as 14-HDHA and 17-HDHA were identified. The level of these precursors was high during the initial phase of acute inflammatory response, decreased during the peak of inflammation, and accumulated in resolution. Based on these results, nanoparticles containing RvD1 or LXA_4_ analogs were constructed and were shown to reduce the influx of granulocytes and to shorten resolution intervals in a mouse model of peritonitis (Norling et al., 2011).

Accumulating evidence account for a role for EVs in the pathogenesis of several neurodegenerative diseases including AD and PD; however, the role of EVs in neuron-glial communication under normal physiological conditions remains under investigated. Deciphering the role of EVs under physiological conditions is crucial for developing therapies to cure or alleviate neurological diseases. For instance, a lipidomic analysis of the composition of brain-derived EVs from patients affected by the different neurodegenerative diseases would be instrumental to identify potential resolution defects associated to a specific pathology and such information could be used to design personalized nanomedicines loaded with specific SPMs (either obtained from EVs extracted from young healthy humans and/or bioengineered) and administrated to patients. However, more information on the specific mechanisms of cell recognition and internalization of EVs by recipient cells as well as how cargoes are unloaded and processed by the recipient cells is required to develop such targeted treatments.

## Concluding Remarks

For a long time it was believed that the acute response to inflammation both in the periphery and in the CNS passively dissipated over time due to a reduced production of inflammatory mediators from either resident non-neuronal cells or infiltrated leukocytes; however, it has been more recently appreciated that also in the brain termination of neuroinflammation is kept in homeostatic balance by the active process of resolution, orchestrated by several families of SPMs, and that ultimately results in the maintenance of brain homeostasis and avoidance of neurodegeneration and subsequent development of neurodegenerative diseases.

Although the biochemical and physiological processes governing the production and biological activities of such pro-resolving mediators and pathways have been largely investigated in peripheral tissues, knowledge on how resolution of inflammation operates in the brain is still at infancy due to many reasons. Firstly, the biggest unresolved issue is to identify the anatomical and cellular source of SPMs within the CNS. Indeed, despite few evidences suggest a local production of SPMs in the brain, probably operated by microglia and astrocytes, as yet it’s not clear which cell type is their actual source, due to the use of mixed cultures, of immortalized cell lines or methods of SPM detection by commercial EIA kits instead of the gold standard liquid chromatography mass spectrometry. As happens in the periphery, where the vascular endothelium is actively involved in the recruitment of leukocytes and in the initial steps of SPM biosynthesis, it is possible that other cells from the brain parenchyma, such as ependymal cells of brain ventricles or choroid plexus, might be involved in SPM production. This is also complicated by their very short half-life and rapid metabolic inactivation, making their endogenous levels challenging to detect in brain cells that are already difficult to culture. The additional difficulty to culture isolated and viable brain-derived cells from mice models or from fresh human biopsies in order to study SPM metabolism and functions make the research in this field even more challenging.

Since astrocytes are natural neuroprotectors and they are the brain cells that express the highest levels of all SPM biosynthesizing enzymes and their target receptors, it is likely that brain resilience can be enhanced by mobilizing the protective potential of these cells through the activation of pro-resolving pathways that indirectly affect neuronal or non-neuronal cells. For instance, the majority of the SPM-mediated potent anti-inflammatory effects observed in microglial cells are likely to be mediated by astrocytes because microglia bear low levels or do not express key SPM receptors such ALX/FPR2, ChemR23, and GPR37. Furthermore, even the SPM-induced protective effects on neurons or oligodendrocytes seem to be indirectly resolved by either astrocytes or microglia, possibly due to the role of SPM in shifting their phenotype toward a pro-resolving and anti-inflammatory one (Table 1 and Figure 2).

**TABLE 1:** Main role of SPMs in astrocytes and oligodendrocytes.

**SPM**	**Receptor**	**Target cell**	**Function**	**References**
LXA4	ALX/FPR2	Astrocytes	Inhibition of IL-8 and ICAM-I Reduction of astrocyte reactivity	Decker et al., 2009; Svensson et al., 2007; Kantarci et al., 2018; Ren et al., 2020
RvD1	ALX/FPR2	Astrocytes	Reduction of astrocyte reactivity Protection of astrocytic mitochondria	Terrando et al., 2013; Bisicchia et al., 2018; Krashia et al., 2019; Ren et al., 2020
RvD2	GPR18/DRV2	Astrocytes	Not reported	–
RvE1	ChemR23/ERV1	Astrocytes	Reduction of astrocyte reactivity	Kantarci et al., 2018
PD1	GPR37	Astrocytes Oligodendrocytes	Not reported Not reported	– –
MaR1	LgR6	Astrocytes	Not reported	–

This not only suggests that a crosstalk between glial cells and neurons occurs also during the process of resolution of inflammation, where cells support and help each other to instate resolutive and protective pathways, but also that dysregulations and defects in resolving inflammation affecting such crosstalk can be involved in the onset and progression of several neuropathologies. In this context, much less is known so far on the exact role of these processes in the different diseases of the CNS. Addressing these questions and how the different brain cells establishing interactions during a resolution process will be a major challenge for the near future and will be helpful to also understand their pathophysiological role and to design better therapeutic strategies aimed at boosting pro-resolving cellular networks rather than breaking off pro-inflammatory ones. This could be achieved by inducing SPM biosynthesis, inhibiting their metabolic inactivation or by synthesizing stable analogs that could be used to treat pathological conditions characterized by exacerbated chronic inflammation and neuroinflammatory diseases.

## Author Contributions

MT and VC designed and wrote the manuscript. Both authors contributed to the article and approved the submitted version.

## Conflict of Interest

The authors declare that the research was conducted in the absence of any commercial or financial relationships that could be construed as a potential conflict of interest.
